# Antifouling Polymer-Coated Anthocyanin-Loaded Cellulose Nanocrystals Demonstrate Reduced Bacterial Detection Capabilities

**DOI:** 10.3390/polym17152007

**Published:** 2025-07-22

**Authors:** Catherine Doyle, Diego Combita, Matthew J. Dunlop, Marya Ahmed

**Affiliations:** 1 Department of Chemistry, University of Prince Edward Island, 550 University Ave., Charlottetown, PE C1A 4P3, Canada; cdoyle2@upei.ca (C.D.); dcombitamerchan@upei.ca (D.C.); 2 Tunistrong Technologies Inc., 99 WB MacPhail Dr., Cornwall, PE C1A 5G6, Canada; matt@tunistrong.com; 3 Faculty of Sustainable Design Engineering, University of Prince Edward Island, 550 University Ave., Charlottetown, PE C1A 4P3, Canada

**Keywords:** antifouling composites, pH-responsive, colour changing, surface grafting, controlled polymerization

## Abstract

Microbial contamination is a global concern with impacts on a variety of industries ranging from marine to biomedical applications. Recent research on hydrophilic polymer-based coatings is focused on combining antifouling polymers with nanomaterials to enhance mechanical, optical, and stimuli-responsive properties, yielding colour changing, self-healing, and super hydrophilic materials. This study combines the hydrophilic and antifouling properties of vitamin B5 analogous methacrylamide (B5AMA)-based polymers with stimuli-responsive anthocyanin-dye-loaded cellulose nanocrystals (CNCs) to develop antifouling materials with colour changing capabilities upon bacterial contamination. Poly(B5AMA)-grafted CNCs were prepared through surface-initiated photoiniferter reversible addition fragmentation chain transfer (SP-RAFT) polymerization and characterized through proton nuclear magnetic resonance (^1^H-NMR), transmission electron microscopy (SEM/TEM), and X-ray photon spectroscopy (XPS) to confirm the formation of surface-grafted polymer chains. The bare CNCs and poly(B5AMA)-grafted CNCs were loaded with anthocyanin dye and evaluated for pH-dependent colour changing capabilities. Interestingly, anthocyanin-loaded CNCs demonstrated vibrant colour changes in both solution and dried film form upon bacterial contamination; however, limited colour changing capabilities of the composites, specifically in dried film form, were attributed to the enhanced dispersibility and antifouling capabilities of the polymer-coated CNCs.

## 1. Introduction

Microbial contamination and biofilm formation are global concerns that affect industries ranging from health care, food production, water treatment plants, and the marine industry [1,2,3]. The modification of surfaces with polymer nanoparticles and pH-responsive dyes to deter bacterial attachment and monitor bacterial contamination over time are widely explored strategies to prevent biofilm formation [1,4,5]. Among the various polymers and nanoparticles studied for their antifouling properties, cellulose nanocrystals (CNCs) present a biodegradable option and a well-studied filler to improve the mechanical properties of polymeric coatings [6,7,8,9,10,11,12,13,14,15]. Recent studies have demonstrated that surface coating of CNCs with antifouling polymers through living radical polymerization can improve the antifouling efficacy of polymeric composites with reduced bacterial and protein attachment in vitro [11]. CNC-encapsulated stimuli-responsive dyes are also shown to detect microbial contamination in food industry and health care settings. For example, CNCs incorporating anthocyanin embedded in polymeric networks demonstrate a visible colorimetric response in the presence of volatile ammonia, and they have been used to monitor the freshness of seafood [16]. Similarly, anthocyanin-loaded CNCs combined with inorganic nanoparticles were shown to provide a visible response to bacterial contamination, yielding bacterial detection capabilities in health settings [17]. The combination of antifouling polymers with food-grade, pH-responsive, dye-encapsulated CNCs has the potential to yield new antifouling materials with improved mechanical and antifouling properties that have not yet been explored. Compared with other reports that physically blend CNCs with polymers, surface functionalization with antifouling polymers improves the solubility and homogeneity of the composites^9^. Furthermore, CNCs have limited adhesion capability and easily peel off of the surface, and so modification of CNCs with polymers and nanoparticles is typically used to develop adhesive biodegradable coatings with various applications [13].

Herein, we combine the hydrophilic and antifouling properties of vitamin B5 analogous methacrylamide (B5AMA) polymers with stimuli-responsive dye-loaded CNCs to develop antifouling materials and evaluate the colour changing capabilities of composites upon bacterial contamination. Poly(B5AMA)-grafted CNCs are prepared through surface-initiated photoiniferter reversible addition fragmentation chain transfer (SP-RAFT) polymerization, and the conversion of the monomer into surface-grafted polymeric chains is analyzed through nuclear magnetic resonance (^1^H-NMR). Compared with other grafting methods that require the separation and purification of free polymer chains, surface-initiated polymerization selectively grafts polymer chains onto the surface of CNCs, eliminating the need for tedious separation of free polymers at the end of the reaction [12]. The poly(B5AMA)-grafted CNCs obtained were analyzed for their composition through X-ray photon spectroscopy (XPS) and for their size through transmission electron microscopy (TEM). The purple-cabbage-derived, anthocyanin-dye-loaded bare CNCs and polymer-grafted CNCs were evaluated for their pH-responsive colour changing capabilities in solution and upon bacterial contamination. CNCs and polymer-modified CNC-coated glass surfaces were then prepared using the brush coating method and analyzed for their surface morphology and scratch resistance through scanning electron microscopy (SEM) and the pencil scratch assay, respectively. The stimuli-responsive capabilities of anthocyanin-loaded, CNC-coated surfaces were analyzed upon contamination with Gram-positive and Gram-negative bacterial strains, and the colour changing capabilities of the coating were monitored using a red chromatic shift (RCS) assay.

## 2. Materials and Methods

### 2.1. Materials

Cellulose nanocrystals (CNCs) were obtained from Tunistrong Inc. (Charlottetown, PEI, Canada). as 10% suspension for wood-pulp-derived CNCs [18]. Prior to polymer modification, CNCs were dialyzed against 3.5 kDa membranes for 72 h and freeze-dried. Diethyl ether, Ethylenediamine (EDA), Methacrylic anhydride, 3-(Trimethylsilyl)-1-propanesulfonic acid sodium salt (TMPS, 97%), Pantolactone, Fluorescein-5-maleimide diacetate, 4,4′-Azobis(4-cyanovaleric acid) (ACVA, 98%), Sodium borohydride (NaBH4, 99%), Carbon disulfide (99.9%), 3-amino(propyl)trimethoxysilane (APTES 99.9%), N,N-Dimethylformamide (DMF, 99.9%), Hydroquinone, Phenol (99%), Ninhydrin, 4-Dimethylaminopyridine (DMAP), Sodium Hydroxide (97%), PBS tablets, Sodium chloride, Sodium carbonate, Sodium bicarbonate, Tween-20, and DMSO were purchased from Sigma Aldrich (Oakville, ON, Canada). Methanol (99.9%), Hexanes (99%), N-Ethyl-N′-(3-dimethylaminopropyl)carbodiimide hydrochloride (EDC•HCl), Mueller Hinton broth (MHB), Ethanethiol, Ethyl acetate (99.5%) 1,4-Dioxane (99%), Acetone, n-butanol (99.7%), Glacial acetic acid, Potassium cyanide (97%) (KCN), Magnesium sulfate anhydrous, Iodine (99.5%), Pyridine, Triethylamine (TEA), Methylene chloride (DCM), 2-β-Mercaptoethanol, Sodium thiosulfate, sodium acetate (99%), potassium chloride (99%), Nutrient Broth, and Isopropanol were purchased from Fisher Scientific.

### 2.2. Preparation of Poly(B5AMA)–CNC Nanocomposites

To prepare the polymeric composites of CTA–CNCs, B5AMA monomer, 90/10 water/methanol solvent, and 100 mg of TMPS (NMR standard) were weighted into a two-necked reactor. CTA–CNC concentration was held constant at 10 mg/mL, and B5AMA monomer was added for a targeted Dp of 10. The mixture was then allowed to stir for 15 min or until good dispersion of the CTA–CNCs was obtained. The reactor was then connected to a Schlenk line at one neck, and a rubber septum was placed on the other neck. A needle connected to nitrogen gas was then inserted into the rubber septum, and the gas flow was adjusted until a steady rate of bubbling was observed. Magnetic stirring was maintained throughout. Nitrogen bubbling was maintained for 30 min, at which time the needle was removed and the reactor placed into a photoreactor, as described in Combita et al., and allowed to polymerize under blue light for 24 h [19].

Percent conversion of the polymerization reactions was monitored through proton NMR. To prepare the NMR sample, prior to connecting the reactor to the Schlenk line, 100 µL of reaction mixture was removed and placed into a 1.5 mL centrifuge tube. Upon completion of the reaction, an additional 100 µL of the reaction mixture was removed and placed in a separate 1.5 mL centrifuge tube. The two samples were centrifuged at 4000 rpm for 10 min. Then, 60 µL of supernatant from each sample was removed and combined with 540 µL of D_2_O.

### 2.3. Extraction of Anthocyanin Dye

Extraction of anthocyanin dye was conducted based on the method of Chen et al. [20]. First, 5 g of freeze-dried ground purple cabbage was placed in 100 mL of 85/15 *v*/*v* ethanol/HCl and left to stir for a minimum of 12 h in the dark. The mixture was then filtered through vacuum filtration. The solid cabbage was discarded, while the filtrate was retained and extracted 3 times with DCM and water. The aqueous phase was retained and placed under rotary evaporation first at 40 °C then up to 55 °C. Once the sample was concentrated to 30 mL, the concentrate was placed into a glass bottle and stored at −20 °C.

### 2.4. Quantification and Evaluation of Dye Extract

Anthocyanin (Cyanidin-3-glucoside) dye extract was quantified using the pH differential method [20], and 0.025 M potassium chloride (pH = 1) and 0.4 M pH 4.5 sodium acetate buffer were used. First, 0.2 mL of dye extract was added to 6 mL of each buffer in separate glass vials. The absorbance of each solution was obtained at 520 nm and 700 nm using an Agilent 8453 UV-Visible spectrophotometer (Santa Clara, CA, USA). The resulting absorbance values were used in Equation (1) to determine the anthocyanin pigment concentration of the sample as a function of cyanidin-3-glucoside equivalents in mg/L. For all samples, if turbidity was observed, the samples were filtered using a simple pipette/kimwipe filter prior to analysis through UV-Vis.

(1) $$Anthocyanin\;dye\;\left(\frac{mg}{L}\right)=A\times MW\times DF\times 10^{3}/\in \times \;l$$

where  A = (A_520nm_ − A_700nm_) pH 1.0 − (A_520nm_ − A_700nm_) pH 4.5.

MW = molecular weight of anthocyanin (cyanidin-3-glucoside) = 449.2 g/mol, DF = 1 is the dilution factor, indicating that the anthocyanin dye extracted was used for the assay and not diluted, l = path length, and ∈ = 28,900 molar extinction coefficient [20].

### 2.5. Dye Loading

First, 60 mg of CNCs or poly(B5AMA)–CNC composites was added to 5 mL of concentrated dye extract and stirred in the dark at room temperature for 48 h. The reaction mixture was then centrifuged (4000 rpm for 10 min), and the supernatant was retained for testing. The pellet was redispersed in a minimal amount of H_2_O and placed in 3.5 kDa dialysis membranes for 72 h with the water changed every 12 h, after which the sample was freeze-dried. Both dialysis and the freeze-drying steps were conducted in the dark.

To evaluate the amount of anthocyanin dye loaded into the CNCs and poly(B5AMA)–CNC composites, the pH differential method was used to compare the absorbance of the original anthocyanin solution to the anthocyanin content of the CNC supernatant after dye loading, as indicated in Equation (1).

### 2.6. Scanning Electron Microscopy (SEM)

The surface topography of the freeze-dried films was studied using SEM (Hitachi, Toronto, ON, Canada). The CNCs and poly(B5AMA)–CNC composites were suspended in a 50/50 *v*/*v* deionized water/ethanol mixture at a concentration of 10 mg/mL and applied to the surface of SEM aluminum stubs with a square paint brush. Then, the stubs were left overnight at room temperature to form a film on the surface. To dry this film completely, the stubs were cooled in liquid nitrogen and then freeze-dried. The mounted samples were then sputter coated with gold and palladium to a thickness of approximately 300 angstroms using a Denton Vacuum Desk II sputter coater (Cherry Hill, NJ, USA). Finally, the coated specimens were digitally imaged using a Hitachi TM3000 SEM and imaging software (Hitachi, Toronto, ON, Canada).

### 2.7. X-Ray Photoelectron Spectroscopy (XPS) Analysis

The XPS measurements were performed on a PHI VersaProbe IV spectrometer (Mississauga, ON, Canada). Measurements were acquired for freeze-dried unmodified CNCs and poly(B5AMA)–CNCs mounted on double-sided adhesive tape. The chamber pressure during measurements was lower than 5 × 10^−7^ Pa. A monochromatic Al Kα source (hν = 1486.6 eV) was used at a power of 25 W. The analysis spot was 100 × 100 µm^2^. The energy step size was 0.8 eV (survey scan) and 0.1 eV (high resolution). The survey scans were collected with an analyzer pass energy of 224 eV. The high-resolution spectra were run with a pass energy of 55 eV. The data were calibrated for a C1s peak at 284.8 eV.

### 2.8. Transmission Electron Microscopy (TEM) Analysis

The morphologies of the unmodified CNCs and the poly(B5AMA)–CNC composites were analyzed using a Hitachi 7700 microscope (Hitachi, Toronto, ON, Canada) with a LaB6 filament operating at an accelerating voltage of 80 kV. The samples were sonicated for 15 min before casting one drop on the 200 mesh copper TEM grids. Excess of the sample was removed and then allowed to dry. The stain, uranyl acetate solution, was then applied. The grid was allowed to dry at room temperature for 24 h before imaging. Image J software v 1.54g was used to measure the diameter and length of the CNC particles [21].

### 2.9. Fourier Transform Infrared Spectroscopy

Fourier transform infrared (FTIR) spectra were obtained in the transmittance mode in the range of 4000–500 cm^−1^ at a resolution of 0.36 cm^−1^ using a Bruker Alpha-T spectrometer (Milton, ON, Canada). The spectra were collected at 16 scans per sample and subtracted from a background spectrum. The spectrometer was operated using the transmission sampling option for the glass slides and the ATR sampling option for the CNC samples.

### 2.10. Scratch Assay

The CNCs and poly(B5AMA)–CNC composites were suspended in 50/50 *v*/*v* deionized water/ethanol mixture at a concentration of 10 mg/mL and applied to the surface of glass slides with a square paint brush. The scratch resistance of the coatings was evaluated using the pencil hardness test, performed in accordance with the standard American Society for Testing and Materials (ASTM) D3363 method. Graphite pencils of varying hardness values of 6H to 6B were sharpened with a standard pencil sharpener and maintained at a contact angle of 45°. Constant force was applied while moving over the coated surfaces. Both scratch and gouge hardness were recorded in this work (scratch is characterized as removal of only a small portion of material, while gouge is the removal of all of the material). Images were obtained using a microscope equipped with a Moticam ProS5 Plus camera (VWR, Mississauga, ON, Canada) and assessed visually.

### 2.11. NMR

All NMR spectra were obtained using a Bruker 300 MHz ^1^H-NMR (Billerica, MA, USA) or a 400 MHz Bruker Avance III ^1^H-NMR spectrometer (Billerica, MA, USA). NMR samples were prepared using D_2_O or DMSO-d^6^ as the solvent. For all spectra in which D_2_O was the solvent, the deuterium oxide peak at 4.79 ppm was used as the reference peak, while for those prepared using DMSO-d^6^, the TMPS standard peak at 0.00 ppm was used as the reference peak.

### 2.12. Colour Change upon Bacterial Burden in Solution

*E. coli* and *M. luteus* were grown in Muller Hinton broth. Bacteria were incubated at 37 °C with shaking. All bacteria were grown overnight before being diluted with broth to a OD_600_ value between 0.25 and 0.29 measured using an Agilent 8453 UV-Visible spectrophotometer (CA, USA). The bacteria were further diluted first through 1:25 dilution (0.4 mL of the sample in 9.6 mL of PBS) and then 1:20 dilution (0.5 mL of PBS sample in 9.5 mL) of Muller Hinton broth. The diluted sample was then used for the experiments.

First, 750 µL of normal saline was placed into each well of a 24-well plate. In sample wells, 150 µL of dilute bacteria was added, while in the control wells, 150 µL of sterile broth was added. To the sample wells and to the control wells, 6 mg of sample (anthocyanin-loaded CNCs and poly(B5AMA)–CNC composites) was added. Additionally, one well per bacterial sample was included without any samples (CNCs and composites) as a control. The plate was then gently agitated and left covered in tinfoil, and it was inspected for colour change every 24 h for up to 96 h or until a clear colour change was observed.

### 2.13. Colour Change upon Bacterial Burden in Films

A solution of anthocyanin-loaded CNCs was prepared in a 50/50 *v*/*v* water/ethanol mixture at a concentration of 30 mg/mL. This mixture was allowed to stir at room temperature for 48 h to ensure a homogenous mixture. Using a square paint brush, the mixture was applied to round glass surfaces in three separate layers with 24 h of drying time between the layers. The coated surfaces were used for the film study.

*E. coli* was grown in 14 mL of Muller Hinton broth and incubated at 37 °C with shaking for 36 h. After 36 h, the bacterial solution was diluted with the broth to an OD_600_ value between 0.6 and 0.65 measured using an Agilent 8453 UV-Visible spectrophotometer. The bacteria were then centrifuged for 5 min at 3800 RPM and at 4 °C. The pellet was retained and redispersed in 10 mL of normal saline. The centrifugation process was repeated on the saline sample, after which the pellet was redispersed in 10 mL of normal saline and placed in a sterile container.

Each coated glass slide was then dip coated into the bacterial suspension. Excess liquid was removed by tapping on the edge of the container, and the slide was then placed into the wells of a 12-well plate. Control samples were dipped in sterile normal saline solution. To control the humidity, the interspace of the plate was filled with normal saline. The plate was then left in the dark at room temperature for 7 days. Photos were taken using an iPhone 11 camera (Apple, Toronto, ON, Canada) against a consistent white background at time 0 and again every 24 h for up to 7 days.

### 2.14. Quantification of Colour Change Using Image J

Quantification of colour change was performed using the method detailed by Weston et al. [22] Photos of the 24-well plates were taken using an iPhone 11 camera with default settings under consistent lighting from a distance of 12 inches from the camera to the plate against a white background. Photos were taken at time 0, time 24, time 48, and time 96 h. Images were then processed to focus on the center of each well. The image size was kept consistent at 150 × 150 pixels. The images were then split into red, green, and blue channels using Image J software. From the RGB histogram of each split, the mean value was taken and used in Equation (2) to determine the red intensity in each pixel. All samples were evaluated in triplicates.

(2) $$r=R/\left(R+G+B\right)$$

The red intensity in each pixel is expressed in terms of red chromaticity (r) and calculated using the mean value from the red split (R), the mean value from the green split (G), and the mean value from the blue split (B).

The red chromaticity values were then applied to Equation (3) to calculate the red chromatic shift percentage (RCS)

(3) $$RCS\%=\left(r_{sample}-r_{0}\right)/\left(r_{max}-r_{0}\right)\times 100\%.$$

RCS was calculated by comparing the red chromaticity at neutral pH, where r_0_ is the red chromaticity of the anthocyanin solution at pH 7, with the red chromaticity of the anthocyanin sample of interest (r_sample_), and r_max_ is the red chromaticity of anthocyanin solution at pH 2.

## 3. Results and Discussion

### 3.1. Poly(B5AMA)–CNC Composite Synthesis and Characterization

**Scheme 1:**
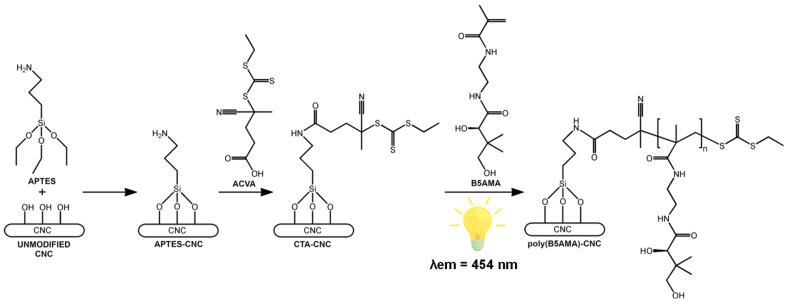
Schematics depicting the “Grafting from” method for surface functionalization of the crystalline nanocellulose (CNC) with poly(B5AMA) through surface-initiated photoiniferter RAFT polymerization.

CNCs prepared through sulfuric acid hydrolysis of wood cellulose were surface modified with poly(B5AMA) in a step-wise approach (Scheme 1). The bare CNCs were first treated with 1 wt.% (3-Aminopropyl)triethoxysilane (APTES) to create amino groups that served as grafting points for the chain transfer agent (CTA) to control the growth of the polymers on the surface of CNCs. The APTES-functionalized CNCs were analyzed qualitatively through Kaiser assay and quantitatively through ortho-phthalaldehyde (OPA) assay [23], indicating substitution of >99% APTES on the CNCs’ surface, and the degree of substitution was determined to be 0.01 (Supporting information Figure S1) [24,25].

4-Cyano-4-(ethylthiocarbonothioylthio)pentanoic acid, a CTA previously documented to yield monodisperse poly(B5AMA) of controlled molecular weight in solution polymerization [19,26], was synthesized, and the synthesis and purity of the compound were confirmed through ^1^H-NMR (Supporting information Figures S2 and S3). APTES-functionalized CNCs were modified with the 4-Cyano-4-(ethylthiocarbonothioylthio)pentanoic acid through carbodiimide chemistry, and functionalization of the CTA on the CNCs’ surface was analyzed through ^1^H-NMR. Consistent with previous reports, modification of CNCs with CTA did not lead to a significant difference in the NMR spectra of the modified CNCs [27]; as such, the indirect fluorophore coupling approach was utilized to determine the amount of CTA grafted onto the surface of CNCs. Because fluorophore can physically adsorb onto the surface of CNCs, extensive washing of the conjugates in organic solvent followed by dialysis in an aqueous solvent was performed to remove any residual amount of unattached fluorophore. The hydrolyzed CTA-grafted CNCs were modified with maleimide-labelled fluorophore, and grafting efficiency was determined by measuring the fluorescence of the modified CNCs in solution using a fluorophore calibration curve (Supporting information Figure S4). The CTA grafting efficiency on the CNCs’ surface was determined to be 5.7 mole% of APTES, and the degree of substitution was calculated to be 0.0004 using Equation (S1) (see the methods section in the supporting information). The degree of substitution is largely dependent on the method used for the modification of CNCs [28]. In this study, the relatively lower degree of substitution may be attributed to the comparison of moles of CTA with the bulk density of hydroxyl groups of CNCs. Because only surface hydroxyl groups of CNCs participate in grafting reactions, the exact number of hydroxyl groups on the CNCs’ surface cannot be determined due to the polydispersity of the samples.

CTA-modified CNCs were grafted with poly(B5AMA) through SP-RAFT polymerization using visible light as the source of CTA activation [19]. The blue light (λ_em_ = 454 nm) exposure activates CTA through n→π* thiocarbonyl absorption, initiating the photoiniferter polymerization reaction and yielding monodisperse polymers of predetermined molecular weights. Compared to thermal polymerization, SP-RAFT polymerization ensures the grafting of polymer chains onto the CNCs’ surface without the need for further purification and separation of free polymer chains from CNC composites in solution [19].

SP-RAFT polymerization was optimized to achieve the highest conversion of B5AMA monomer to the grafted polymer chains by evaluating CNCs’ dispersibility as a function of concentration and reaction solvent type, as solution clumping of the CNCs was detrimental to the surface grafting of poly(B5AMA). The polymerization reactions performed in water/organic solvent mixtures, including MeOH, DMSO, and dioxane as organic components, were evaluated, and the percent conversion of B5AMA monomer to CNC-grafted polymer chains was determined through the comparison of the integral values of the peak at 5.8 ppm with the TMSPS reference peak at 0 ppm of NMR spectra (Supporting information Figure S5). The polymerization reactions performed in DMSO- and MeOH-based solvents with a targeted Dp of 10 showed superior polymer grafting efficacies (10–11% of the monomer conversion into grafted polymer chains), whereas no conversion was obtained for the reactions performed in the dioxane:water mixture (Supporting information Table S1). The surface grafting of polymer chains through photoiniferter polymerization reaction was further optimized as a function of the concentration of CNCs in a 90/10 *v*/*v* water/MeOH mixture, and the highest conversion of B5AMA to grafted polymers (12 ± 4%) was obtained at a concentration of 10 mg/mL of CTA-functionalized CNCs with a targeted Dp of 10 (Table 1, Supporting information Table S1). Kavand et al. investigated the formation of hyperbranched polymers on the surface of up-conversion nanoparticles through SI-RAFT and demonstrated the conversion of monomer into polymers in the range of 7% to 18%, as determined through proton NMR [29]. Further increases in the degree of polymerization reduced the overall percent conversion of the monomer into CNC-grafted polymer chains, possibly due to the steric hindrance of large growing chains on the surface of CNCs (Supporting information Table S2).

**Figure 1:**
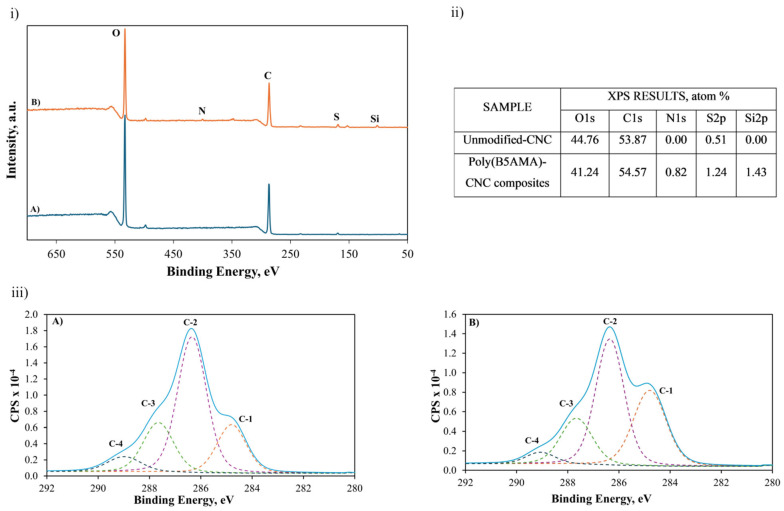
X-ray photoelectron spectroscopy (XPS) analysis of unmodified CNC and poly(B5AMA)–CNC composite: (**i**) survey scan of unmodified CNC (**A**) and poly(B5AMA)–CNC composites (**B**); (**ii**) atomic composition of unmodified CNC and poly(B5AMA)–CNC composite; (**iii**) C1s peak deconvolution of unmodified CNC (**A**) and poly(B5AMA)–CNC composites (**B**).

**Figure 2:**
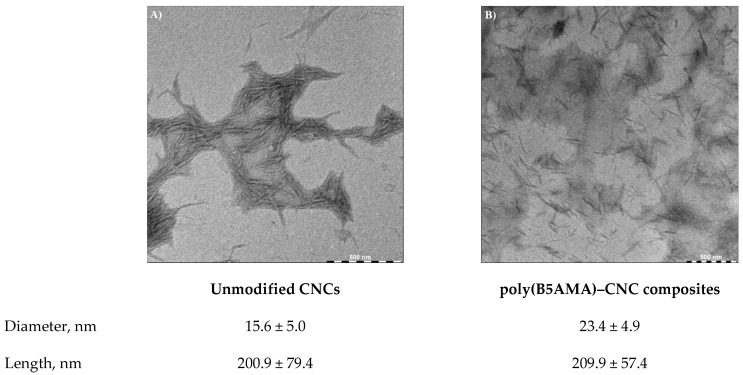
TEM images of the unmodified CNCs (**A**) and the poly(B5AMA)–CNC composites (**B**).

X-ray photoelectron spectroscopy (XPS) was used to confirm the functionalization of CNCs with APTES, CTA, and poly(B5AMA) (Figure 1i). In addition to the peaks at 533 eV (O1s) and 286 eV (C1s), unmodified CNCs showed a peak at 169 eV (S2p), corresponding to the sulfur content of 0.51 atom%. This small amount of sulfur is associated with the sulfuric acid hydrolysis method used to obtain the cellulose nanocrystals [24]. On the other hand, modification with APTES poly(B5AMA)–CNCs showed additional peaks at 102 and 400 eV, which correspond to silicon (Si2p) and nitrogen (N1s). This change in the composition can be explained by the grafting of poly(B5AMA), where the appearance of silicon and nitrogen is associated with CNC modification using the silanization agent (APTES) and CTA and polymer grafting. In turn, the sulfur content increased from 0.51 to 1.24 atom% for the unmodified- and poly(B5AMA)-modified CNCs, respectively, which is explained by the grafting of the CTA agent, a trithiocarbonate molecule (Figure 1ii). The presence of polymer brushes on the surface of the CNC increased the carbon content measured through XPS, from 53.87 atom% for the unmodified CNC to 54.57 atom% for the poly(B5AMA)–CNC. Indeed, the deconvolution of the C1s signal showed an increase in the peak corresponding to C-1 (C—C and C—H bonds in the polymer graft) (Figure 1iii). On the other hand, the morphology modification of the CNCs after the grafting of poly(B5AMA) was evaluated using TEM. The unmodified CNCs exhibit a needle-like structure with a diameter of 15.6 ± 5.0 nm and a length of 200.9 ± 79.4 nm. The grafting of the polymer did not affect the morphology or the length of the CNC particles (209.9 ± 57.4 nm), although its diameter increased to 23.4 ± 4.9 nm, which confirms the successful grafting of poly(B5AMA) on the CNC’s surface (Figure 2).

### 3.2. Anthocyanin Extraction and Solution Properties of Dye-Loaded CNCs and Composites

Microbial growth is known to influence the pH of the surrounding environment, and it can serve as useful indicator to test the growth of microbes on stimuli-responsive surfaces [25]. Anthocyanin dye extracted from purple cabbage, a commonly used, food-grade, pH-responsive probe with colour changing capabilities, was extracted using the solid–liquid extraction method, and the amount of anthocyanin pigment attributed to cyanidin-3-glucoside equivalent present in the liquid extract was quantified using the pH differential method provided in Equation (1) [20]. The average concentration of anthocyanin dye obtained after purification was 931 ± 18 mg/L, and this was consistent with previously reported values in the literature ranging from 19.3 mg/L to 1187 mg/L of the dye depending upon the cabbage type and the reaction conditions used for dye extraction [27,30].

The stimuli-responsive properties of extracted anthocyanin evaluated using the pH differential method through UV-visible spectroscopy demonstrated vibrant colour change as a function of pH in aqueous solution (Supporting information Figures S6 and S7). The stimuli-responsive CNCs and poly(B5AMA) –CNC composites were then developed by mixing the dye extract with the CNCs, and the amount of dye loaded was evaluated by measuring the difference in anthocyanin concentration in solution before and after incubation with CNCs and the composites. The dye-loaded CNCs and poly(B5AMA)–CNC composites were extensively dialyzed to remove free anthocyanin. The amount of anthocyanin dye loaded in poly(B5AMA)–CNC composites was relatively higher (3.7 µg/mg of composites) than the amount loaded into CNCs (2.7 µg/mg of CNCs), possibly due to the higher dispersibility and reduced clumping of poly(B5AMA)–CNC composites in solution compared with the unmodified CNCs.

The evaluation of chromic transitions of anthocyanin, typically measured using spectroscopy techniques, is typically performed on transparent solutions with limited translational capability towards opaque or colloidal samples, such as CNCs [20]. The colour change capability of anthocyanin-loaded CNCs and their composites in solution was analyzed and quantified through the measurement of a red chromatic shift (RCS) assay. RCS assay is a visual technique that analyzes RGB values of sample images to measure %RCS values. An incremental increase in pH results in a colour change from red to blue, with higher %RCS values associated with lower solution pH and vice versa [20].

The colour changing properties of anthocyanin-loaded CNCs were first measured through the incubation of materials in sterilized media (pH 7–8) and in the absence of bacteria. The addition of anthocyanin-loaded materials in sterilized media demonstrated an instant colour change from bright pink (in powder form) to a greyish colour due to the change in solution pH, indicating that the dye-loaded material maintained the stimuli-responsive properties of anthocyanin. The addition of Gram-negative (*E. coli*) and Gram-positive (*M. luteus*) bacteria in the dye-loaded CNC solution did not cause an immediate colour change; however, a noticeable change from grey to green was observed after 48 h of incubation, indicating a shift towards basic pH due to the byproducts formed during bacterial growth [31].

**Figure 3:**
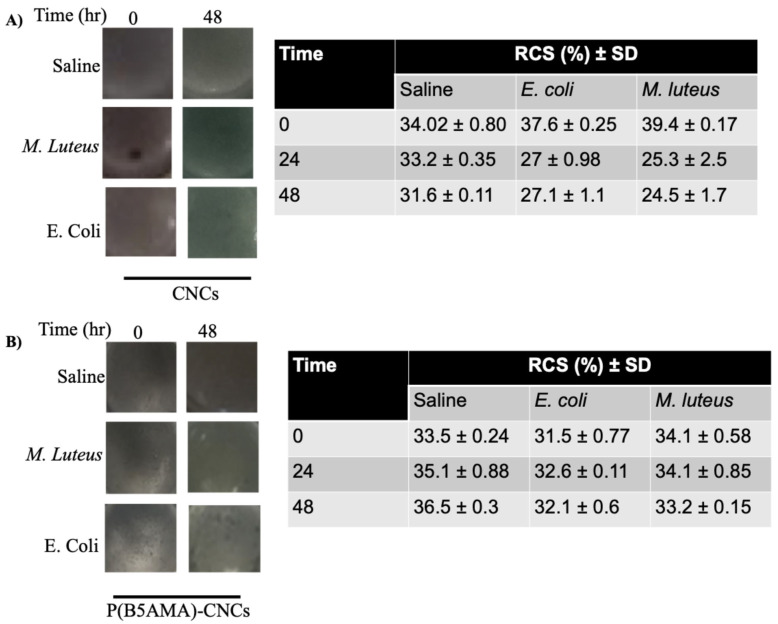
Quantification of colour change in anthocyanin-loaded (**A**) CNCs and (**B**) poly(B5AMA)–CNC composites in response to bacterial burden (*M. luteus* and *E. coli*) measured through red chromatic shift (RCS). n = 3, SD represents standard deviation, at time 0 and time 48 h.

The addition of poly(B5AMA)–CNC composites in sterilized media demonstrated a similar change in colour from bright pink in powder form to grey that was comparatively less pronounced than that of bare CNCs; furthermore, no measurable change in colour compared with the ones in the bacteria-free control was observed 48 h post-incubation in bacterial solution (Figure 3). Further incubation of p(B5AMA)–CNCs in bacterial solution for up to 96 h did not yield any noticeable colour change (Supporting information Figure S8) compared to the untreated control. Bacterial viability in the presence of CNCs and poly(B5AMA)–CNCs was evaluated using a colony counting assay, and no significant change in bacterial viability compared with the untreated control was observed, indicating that the difference in colour change for CNCs and polymeric composites was not due to any significant change in bacterial viability of the two samples (Supporting information Figure S9). Poly(B5AMA) is a well-documented antifouling polymer that prevents the attachment of bacteria and other small molecules, such as proteins, on the grafted surface [19,26]. The reduced colour changing capabilities of anthocyanin-loaded poly(B5AMA)–CNCs may be attributed to the antifouling behaviour of poly(B5AMA)-grafted CNCs, which forms a hydration layer around the composites and minimizes the interaction of bacterial byproducts formed during growth with the dye-loaded CNCs.

### 3.3. Polymeric Composite-Coated Surfaces

**Figure 4:**
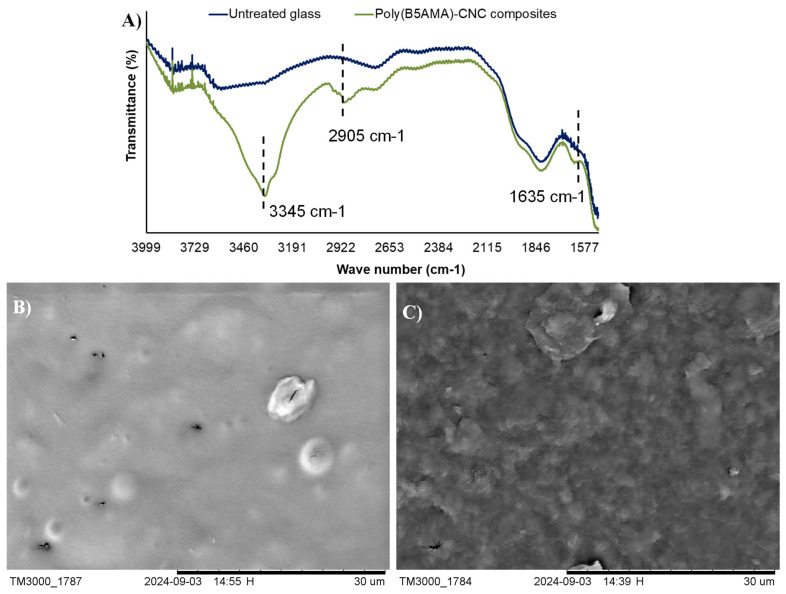
(**A**) FTIR analysis of CNC and poly(B5AMA)–CNC composite coated glass surface. SEM images of (**B**) CNC and (**C**) poly(B5AMA)–CNC composite coated surface prepared using the brush coating method. Samples were prepared in 50/50 *v*/*v* ethanol/water at a concentration of 10 mg/mL.

CNCs and their polymeric composite-coated glass films were prepared through physical coating of the materials onto the glass surface. The slides were dried and analyzed through FTIR spectroscopy. Figure 4A shows the spectra of bare glass and the composites applied onto the glass surface.

The bare glass absorbs strongly below 1500 cm^−1^, and presence of the CNC composite on the glass surface could not be verified at lower frequencies. However, at higher frequencies, the characteristic absorption peaks of both crystalline nanocellulose and poly(B5AMA) at 1635 cm^−1^ (O–H vibration of absorbed water), 2905 cm^−1^ (asymmetric stretching vibration of C–H/C–H2), and 3345 cm^−1^ (O–H stretching of intra- and intermolecular hydrogen bonds) were observed (Figure 4A, Supporting information Figure S10) [31,32,33].

The surface morphology studied through SEM showed the presence of a plain smooth surface for CNCs, while poly(B5AMA)–CNC coatings showed increased surface roughness, possibly due to the loss of CNC alignment patterns [34] upon polymer grafting and the agglomeration of composites during the drying process (Figure 4B,C). However, consistent with previous reports, bare CNC coatings were very flaky and easily peeled off during SEM analysis [13]. In contrast, poly(B5AMA)–CNC coatings were highly adhesive and stable.

The strength of CNCs and Poly(B5AMA)–CNC coatings prepared on glass surfaces were evaluated using the pencil scratch assay. The poly(B5AMA)–CNC-coated glass surface showed a clear improvement in gouge hardness (F) and scratch hardness (HB) compared with the CNC-coated surface, with scratch hardness of 3B and gouge hardness of 2B (Table 2, Supporting information Figure S11).

**Figure 5:**
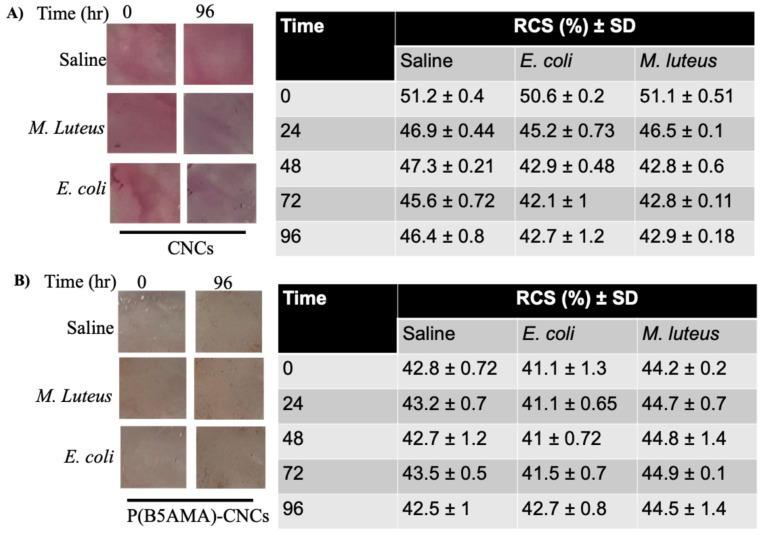
Quantification of colour change in (**A**) anthocyanin-loaded CNC and (**B**) the composite-coated surfaces in response to bacterial burden (*M. luteus* and *E.coli*) through red chromatic shift (RCS). n = 3, SD represents standard deviation, at time various time points.

The dye-loaded CNCs formed a bright pink-coloured coating, and our preliminary experiments indicated that a minimum of three coats of anthocyanin-loaded CNCs on bare glass was sufficient to visually detect the colour change of anthocyanin dye upon bacterial contamination. The anthocyanin-loaded, CNC-coated glass slides were exposed to Gram-positive and Gram-negative bacteria (*M. luteus* and *E*. *coli*), and the colour change of dried films was observed for up to 5 days and compared with the sterile saline treated control. As seen in Figure 5A, exposure to bacteria caused a clear colour change from pink to purple compared with the untreated control for the CNC-coated glass surface. The bacterial detection ability of the anthocyanin-loaded CNCs observed in solution form was maintained in dried film form, indicating the versatility of the material developed as a pH sensor for bacterial growth detection [16,17].

Despite their superior dye loading capabilities, p(B5AMA)–CNC composite-based films demonstrated a weak and rather insignificant colour of anthocyanin dye in the form of dried films. The addition of bacteria did not restore the colour responsiveness of the composites, and no measurable colour change was observed upon bacterial contamination for up to five days. The difference in colour change capabilities of p(B5AMA)–CNC composites in solution and dried film form may be partially attributed to the antifouling properties of the polymer and the difference in CNCs’ alignment in bare and polymeric composite form. Bare CNCs strongly clump and align themselves in dried form, possibly contributing to the vibrant hues of the dried films, whereas polymeric composites are more dispersed, forming a rough and non-uniform layer and hence limiting the colour yielding capabilities of dye-loaded composites [34].

## 4. Conclusions and Future Directions

Cellulose nanocrystals were successfully modified with the antifouling polymers of vitamin B5 analogous methacrylamide through surface-initiated photoiniferter RAFT polymerization. The surface grafting of polymers on CNCs was confirmed through ^1^H-NMR, XPS, and TEM analysis. pH-responsive anthocyanin dye was extracted from purple cabbage and tested for pH-responsive capabilities using the pH differential method. The dye-loaded bare CNCs and the polymeric composites prepared were quantified for dye encapsulation efficacies. Our results demonstrated that poly(B5AMA)-grafted CNCs demonstrated superior anthocyanin encapsulation efficacies compared to bare CNCs, possibly due to the enhanced water dispersibility of the composites. As expected, dye-encapsulated CNCs specifically demonstrated bright pink hues in acidic solution that instantly changed to a greyish colour in pH 7.5 bacterial culture media. The anthocyanin-loaded pH-responsive CNCs demonstrated colour changing properties in response to bacterial insult in solution within 48 h of incubation. However, the results were perplexing for polymeric composites, as, despite superior dye loading efficacies, the polymeric composites in general showed weakly coloured solution and poor response to bacterial insult. The poly(B5AMA)-grafted CNCs demonstrated insignificant colour change even after seven days of incubation with the bacteria. The evaluation of the mechanical properties of surfaces demonstrated the superior scratch resistance of poly(B5AMA)–CNC-coated surfaces compared to bare CNC coatings. As expected from the solution properties, anthocyanin-loaded, CNC-coated films demonstrated a vibrant pink colour in a dehydrated state, and exposure to bacterial droplets resulted in a clear change from a pink to a purple colour. However, consistent with the colour change analysis in solution, anthocyanin-loaded CNC composites showed limited colour changing capabilities in film form, possibly due to the antifouling capabilities of the poly(B5AMA). The %RCS values calculated from surface images demonstrated no significant change in colour after days of incubation of bacteria on the polymeric composite films. This limited colour changing response of composites in the presence of bacterial insult was attributed to the limited interactions of bacterial byproducts with antifouling polymer-coated surfaces or the reduced clustering and alignment of polymer-coated CNCs in solution and dried film form, producing weak hues of the encapsulated dye. Further studies will be required to understand the differences in colour changing capabilities of antifouling polymeric composites compared to the bare CNCs in both solution and film form and to understand their potential as sensors for bacterial detection.

## Figures and Tables

**Table 1:** Degree of substitution (DS) of CNCs with APTES and CTA and polymerization reaction parameters conducted using 90/10 *v*/*v* water/methanol as the solvent for a targeted Dp of 10 and at a CTA–CNC concentration of 10 mg/mL.

Samples	Targeted Molecular Weight (Da)	DS of APTES	DS of CTA	B5AMA Conversion (%)	Anthocyanin (µg/mg of CNCs)
w-CNCs	-	-	-	-	2.7 ± 0.3
P(B5AMA)–CNCs	2600	0.01	0.0004	12 ± 4	3.7 ± 0.3

**Table 2:** Softer < 6B-5B-4B-3B-2B-B-HB-F-H-2H-3H-4H-5H-6H > harder.

Glass Surface Coated with	Surface Hardness Pencil Scale Scratch Gouge	
w-CNCs	3B	2B
Poly(B5AMA)–CNCs	HB	F

## Data Availability

The original contributions presented in this study are included in the article/Supplementary Materials. Further inquiries can be directed to the corresponding author.

## Supplementary Materials

The following supporting information can be downloaded at https://www.mdpi.com/article/10.3390/polym17152007/s1. References [19,23,26,35,36,37,38] are cited in the supplementary materials.
