# Supplementary material for: Antifouling Polymer-Coated Anthocyanin-Loaded Cellulose Nanocrystals Demonstrate Reduced Bacterial Detection Capabilities
Source: Polymers (Basel). 2025 Jul 22;17(15):2007. doi: 10.3390/polym17152007 (PMC12349685; doi:10.3390/polym17152007)
Supplement: Supplementary file 1 [file polymers-17-02007-s001.zip › polymers-3748775-supplementary.pdf]

Supporting Information

Antifouling Polymer Coated Anthocyanin-Loaded Cellulose Nanocrystals Demonstrate Reduced Bacterial Detection Capabilities

Catherine Doyle[1], Diego Combita[1], Ranga Dissanyake[1], Marya Ahmed[1,2]*

Department of Chemistry[1] and Faculty of Sustainable Design Engineering[2]

University of Prince Edward Island

550 University Ave. Charlottetown, PE C1A 4P3, Canada

Corresponding author: **marahmed@upei.ca*

Materials and Methods

Concentrated hydrochloric acid (36.5-38%), and Sodium fluorescein were purchased from Anachemia. Deuterium oxide (D_2O) and Dimethyl sulfoxide-d6 (DMSO-d6) were purchased from Cambridge Isotope Laboratories. o-Phtalaldehyde (OPA) was purchased from TCI Chemicals. Ethanol anhydrous was purchased from Commercial Alcohols. Agar A was purchased from Biobasic. Cultures of *M. luteus* were purchased from Cedarlane. Cultures of *E. coli* were obtained from the Kerr Laboratory at UPEI. Purple cabbage was purchased from a local grocery store, and freeze dried immediately upon receipt. 4-Cyano-4-(ethylthiocarbonothioylthio)pentanoic Acid (TCT-2), Vitamin B5 analogous methacrylamide (B5AMA), 2-Aminoethylmethacrylamide hydrochloride (AEMA), and Ethylenediamine dihydrochloride (EDA•2HCl) were synthesized following previously reported procedures.[19,26]

Modification with APTES

Modification of CNCs with APTES was based on the method by Zhang *et al*.[35] 250mg of CNCs were placed in 100 mL of 50/50 v/v ethanol/water and allowed to stir until a homogenous mixture was obtained. In a separate vial, 0.25mL of APTES was placed in 10mL of an 80/20 v/v ethanol/water solvent. This mixture was allowed to stir for 1 hour. After the two solutions were combined, the pH of the mixture was adjusted to ≈ 4 by the addition of glacial acetic acid. The mixture was then left to stir for 2 hours. Following this the sample was dialyzed against 3.5 kDa membrane for 24 hours, then freeze dried. The surface modification was evaluated by Kaiser assay.[36]

Quantification of APTES Modification

An APTES grafting reaction was conducted as per the previously established procedure[26], however prior to dialysis, the reaction mixture was centrifuged (4000rpm for 15 minutes) and the supernatant retained. Amine determination was conducted according to the method described in interchim protocol.[23] An Ortho-Phtalaldehyde (OPA) working solution containing 5 µL of 2-β-mercaptoethanol, 100 µL ethanol, 10 mg OPA, and 10 mL of 50 mM carbonate buffer of pH10.5 was prepared fresh and used within two hours. The

procedure for the analysis was as follows: 100 µL of OPA working reagent and 100 µL of CNCs supernatant, post-functionalization reaction was placed into wells of a 96 well plate in triplicate. Then the absorbance of each sample was measured at 340 nm, exactly 1 minute after the addition of the OPA reagent. The blank for each sample was the absorbance at 340 nm of the supernatant without the addition of OPA. A calibration curve of samples with known APTES concentrations was prepared to allow for quantification of the results by linear regression. Degree of Substitution (DS) upon APTES modification was calculated using the equation 1, using molar mass of anhydrous sugar and APTES 162 and 221.37 g/mol, respectively.

DS = (mass of substituent / molar mass of substituent) / (mass of CNCs / molar mass of anhydrous glucose unit) (1)

Modification with RAFT Agent

Synthesis of the RAFT agent TCT-) involved a two-step process, beginning with the preparation of the disulfide precursor according to the method by Barlow *et al.*[37] Modification of APTES-CNCs with the RAFT agent was conducted according to the method by Chen *et al.*[38] 59 mg of 1% APTES-CNCs were left to stir in 25 mL DMSO until good dispersion was achieved. Then 17.5mg of DMAP was added, following which 68mg of 4-cyano-4-(ethylthiocarbonothioylthio)pentanoic Acid was added. After 10 minutes of stirring, 97mg of EDC•HCl was added with stirring. After this the mixture was placed in an oil bath at 40°C and left for 24 hours under nitrogen atmosphere. After 24 hours the mixture was purified by washing and centrifuging (4000rpm for 10 minutes) with ethanol three times, the pellet was then redispersed in water and freeze dried. The freeze-dried product (referred to as CTA-CNCs) was then stored at -20°C under nitrogen atmosphere.

Quantification of RAFT Agent Grafting

8 mL of 0.5M sodium borohydride solution was added to the RAFT agent grafted CNCs. This mixture was allowed to stir uncapped in a fume hood for 24 hours. Following which the product was dialyzed in 3.5kDa membranes for 72 hours, then freeze dried.

A 3mg sample of the freeze-dried product was retained and used as a blank in the fluorescence studies however the remaining hydrolyzed product was reacted with fluorescein-5-maleimide. 3mg of fluorescein-5-maleimide was added to the product of the sodium borohydride reaction, to which 5mL of DMF was added. The mixture was left to stir in darkness for 24 hours. After 24 hours the mixture was purified by centrifuging (4000rpm for 10 minutes) and washing with DMF. The pellet was then freeze dried in dark.

A calibration curve was prepared using sodium fluorescein in a 1:10 v/v DMF/water solvent. The sample blank contained sodium borohydride hydrolyzed CTA-CNCs. The samples and the blank were prepared at a concentration of 3mg/mL. For the calibration curve the blank was solvent alone. The calibration curve was prepared by serial dilution of fluorescent dye. To obtain the fluorescence intensity the following parameters were used excitation of 494nm and emission of 519nm with a bandwidth of 5nm. A linear regression was performed on the graph with fluorescence on y-axis and concentration of the dye at x-axis, which allowed for the fluorescein concertation of the material to be determined and used as an indirect method of estimating RAFT agent-grafting percentage.

OPA Assay

O-phthalaldehyde (OPA) assay was performed to obtain quantitative analysis of amine functionalization of CNCs.

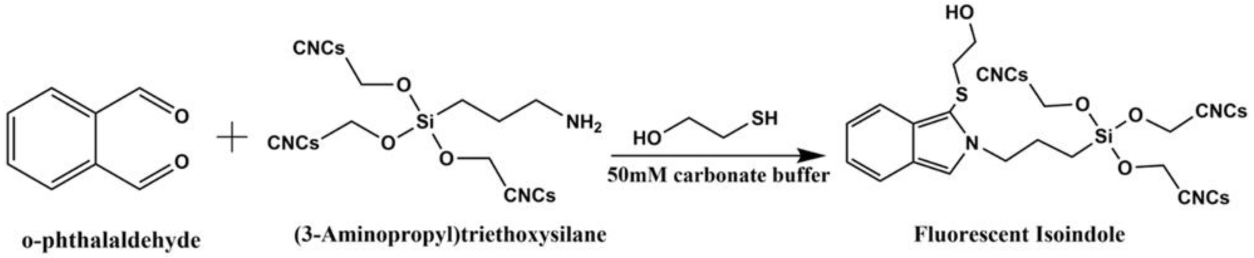

OPA reacts with the primary amine of APTES and mercaptoethanol to produce a fluorescent isoindole, which can then be detected by UV-Vis spectroscopy by measuring absorbance at 340nm.

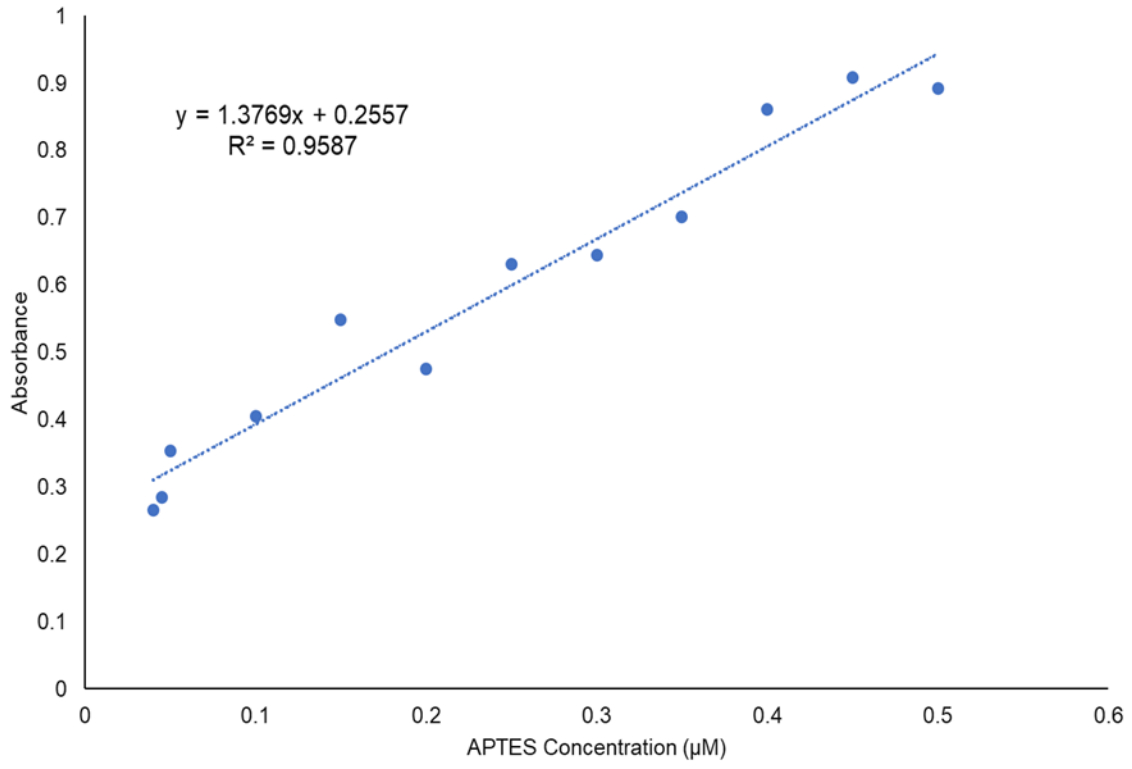

Figure S1: Absorbance at 340nm versus APTES concentration, Calibration curve prepared by APTES detection by OPA amine detection protocol. The relationship between absorbance and APTES concentration was linear with R^2 value of 0.9587. Based on this equation of the linear regression and the absorbance value of the supernatant of the APTES silanization reaction, it was determined that >99% of the APTES was grafted on to the CNCs surface.

Synthesis of the RAFT Agent:

The synthesis of 4-Cyano-4-(ethylthiocarbonothioylthio)pentanoic acid was done by a two-step method. The first step was the synthesis of the disulfide precursor from ethanethiol and carbon disulfide. The second step was the formation of the final trithiocarbonate product from reaction of the disulfide precursor and 4,4′-Azobis(4-cyanovaleric acid) (ACVA).

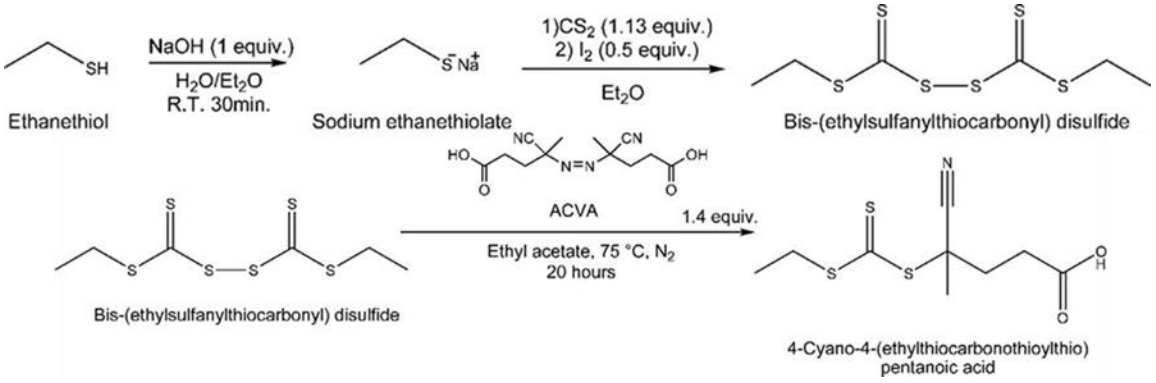

Reaction scheme for the preparation of the CTA 4-Cyano-4 (ethylthiocarbonothioylthio)pentanoic acid referred to as TCT-2.

The ^{1}H NMR spectrum of the disulfide precursor is given in Figure S2 while the ^{1}H NMR of the final product 4-Cyano-4-(ethylthiocarbonothioylthio)pentanoic acid is given in Figure S3.

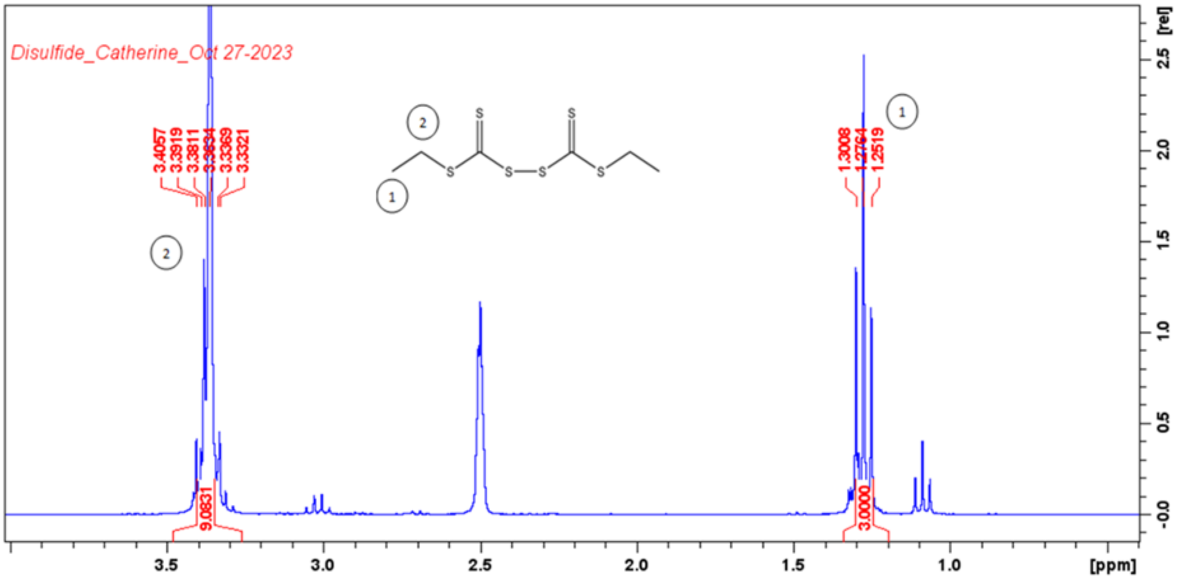

Figure S2: Assigned ^{1}H NMR spectrum of bis-(ethylsulfanylthiocarbonyl) disulfide.

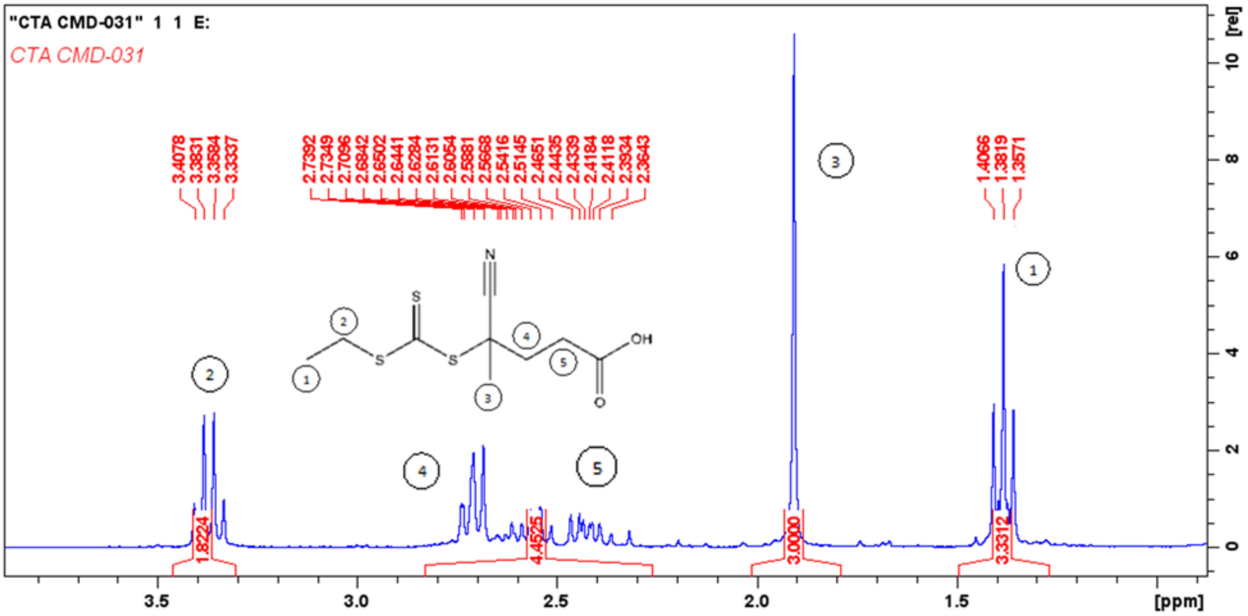

Figure S3: ^{1}H NMR spectrum of the CTA 4-Cyano-4-(ethylthiocarbonothioylthio)pentanoic acid. The yield obtained for the synthesis was approximately 50%. Following synthesis the CTA was stored under nitrogen in a sealed vial in the freezer at -20°C and was used in subsequent grafting reactions.

Modification of APTES-CNCs with the RAFT Agent:

An indirect method for CTA quantification using maleimide chemistry was developed. The first step of this reaction involved hydrolysis of the CTA-CNCs, by a reducing agent, to form thiols on the surface of the CNCs. Following purification, the hydrolyzed-CNCs were reacted with the fluorescein-5-maleimide, where each thiol molecule on the surface of the CNCs would react with 1 molecule of fluorescein-5-maleimide.

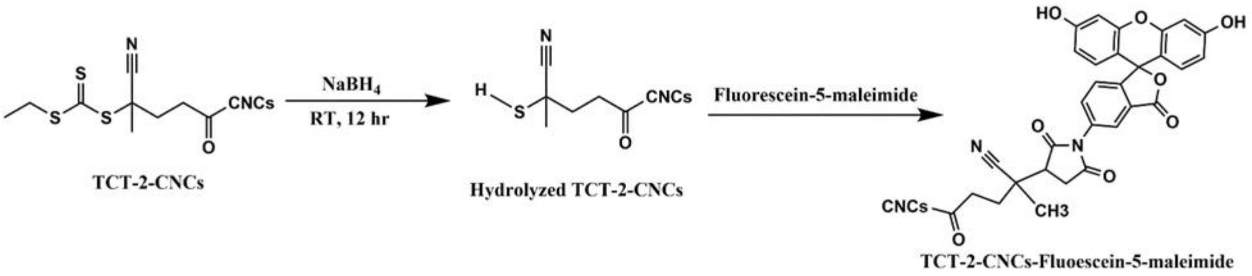

Reaction scheme for TCT-2 determination by maleimide Chemistry.

This method allowed indirect estimation of the amount of CTA present on the surface of the CNCs by measuring the amount of fluorescein on the CNCs using fluorescence spectroscopy. In order determine how much fluorescein-5-maleimide was grafted on to the CNCs, a calibration curve was prepared using sodium fluorescein which is given in Figure S4.

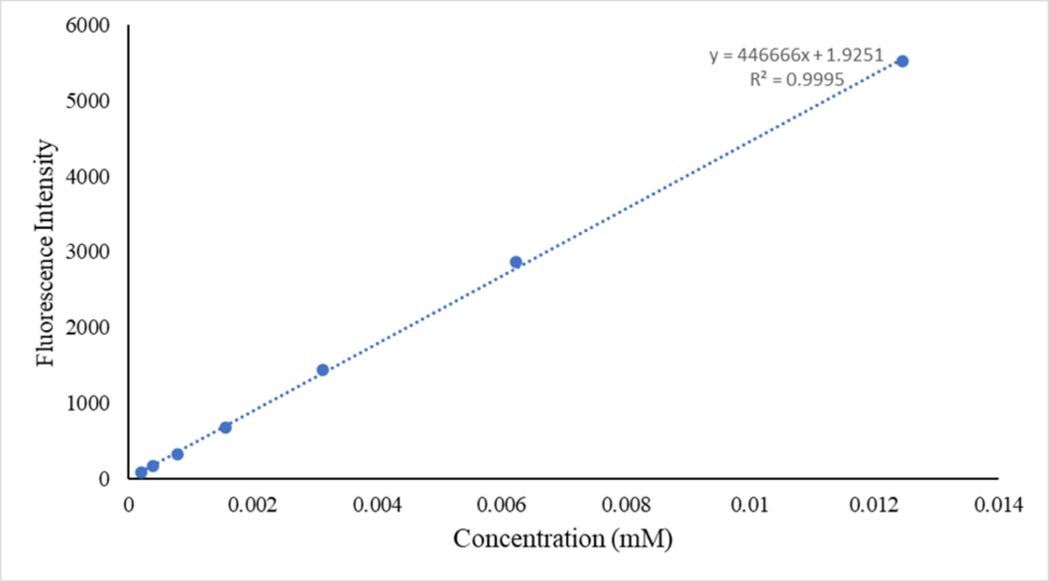

Figure S4: Calibration curve prepared using sodium fluorescein for the determination of fluorescein-5-maleimide grafting to CTA-CNCs, obtained with an excitation of 494nm, and emission of 519nm with bandwidth of 5nm. the relationship between fluorescence and fluorescein concentration was linear with R^2 value of 0.9995. Based on this equation of the linear regression and the fluorescence value of the fluorescein-5-maleimide-CTA-CNCs, it was determined that CTA grafting efficiency was 5.7 mol% of APTES. This value converted into degree of substitution of APTES is 0.057.

Optimization of SI-PI-RAFT Polymerization to Prepare poly(B5AMA)-CNC Composites:

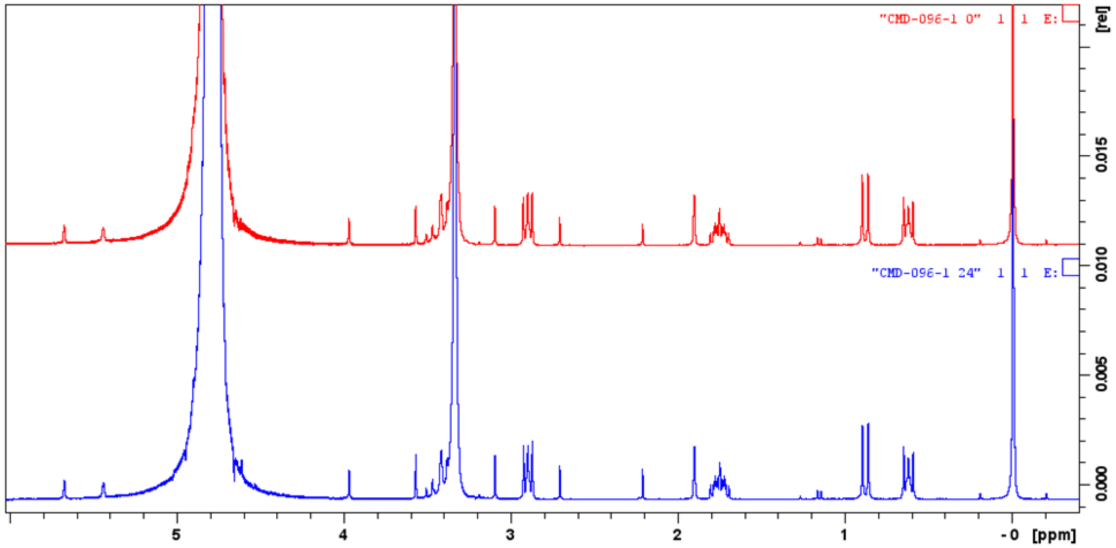

Figure S5: Overlaid ^{1}H NMR spectra for reaction CMD-096-1 at time 24 (blue) and time 0 (red).

Conversion of B5AMA into poly(B5AMA) was calculated by comparison of the integral values of the peak at 5.8ppm and the TMPS peak at 0 ppm. As can be seen in Figure S5, there is a slight decrease in intensity of the peaks at 5.8 and 5.2ppm after the reaction (time 24), which is a result of conversion of monomer to polymer.

Table S1: Optimization of the solvent type for polymerization reaction. The concentration of CTA-modified CNCs was 10 mg/mL in each reaction and targeted Dp was 10.

Reaction Code	Solvent Type	Conversion % by ^{1}H-NMR
CMD-099-2	90/10 v/v water/DMSO	11
CMD-099-3	90/10 v/v water/1,4- dioxane,	0
CMD-100	90/10 v/v water/methanol	10

Table S2: Investigation of the effect of Dp on reaction conversion. Solvent was Water MeOH mixture, 90/10 v/v. The concentration of CTA-CNCs was 10 mg/mL.

**Reaction Code**	**Dp**	**Conversion (%) by ^{1}H-NMR**
CMD-070	50	2
CMD-088	100	3

Evaluation of the Anthocyanin Dye Extract

The anthocyanin extract was evaluated for its pH-dependent colour changing ability. Dilute solutions of the extract were pH adjusted across the range 2-12 and analyzed both visually and by UV-Visible spectroscopy. The samples exhibited a vibrant colour change as the pH values changed in water.

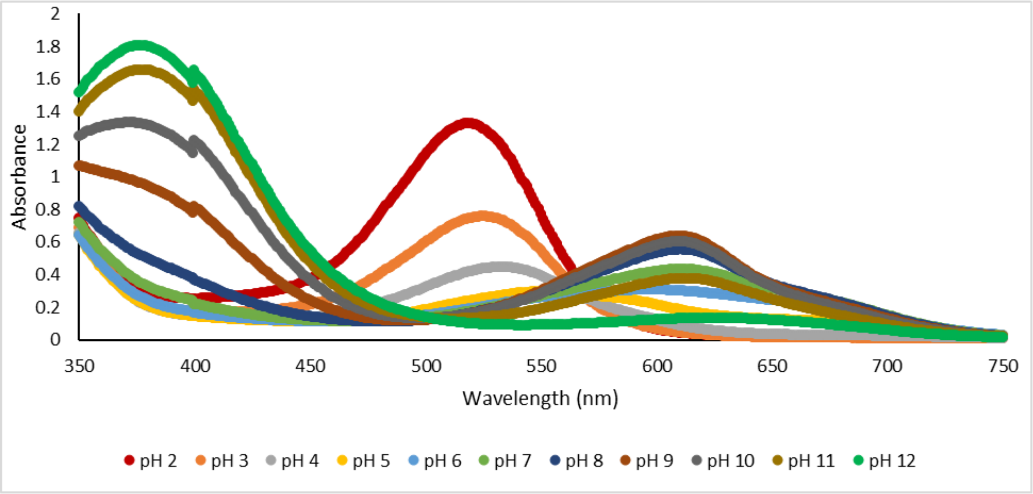

Figure S6: Overlaid absorbance spectra for the liquid anthocyanin extract recorded at various pH.

The wavelength of maximum absorbance is shifted as a result of pH increase, which provides quantitative evidence of the colour change exhibited by the samples. This is supported by the clear visual colour change shown in Figure S7.

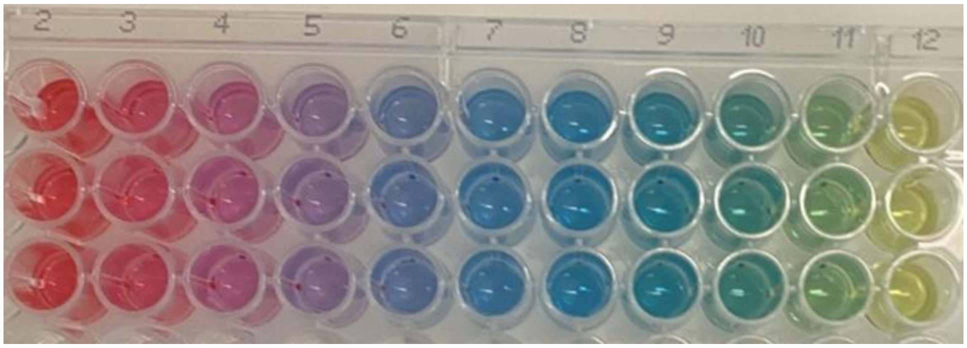

Figure S7: Image of liquid dye extract at pH values ranging from 2 (left) to 12 (right).

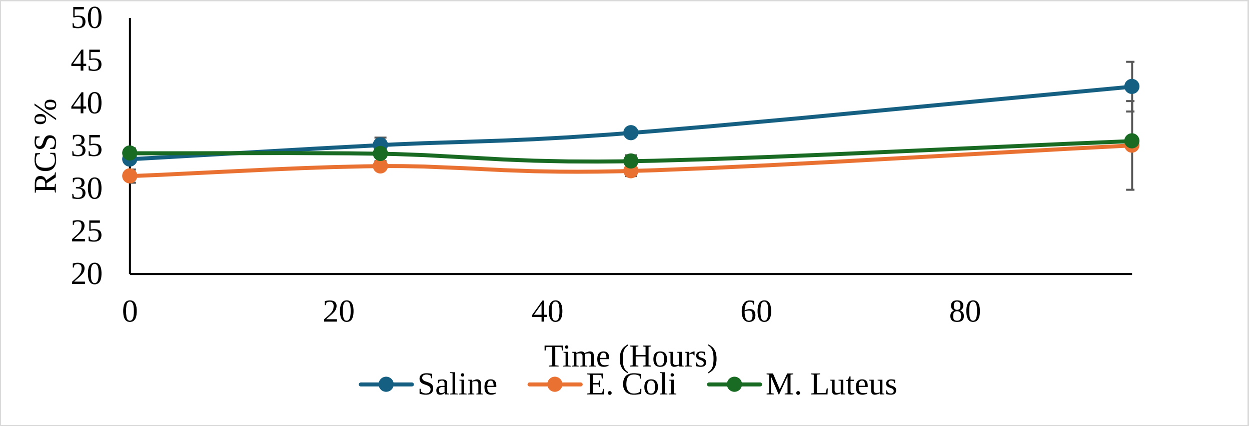

Figure S8: Quantification of color change in anthocyanin loaded poly(B5AMA)-CNC composites in response to bacterial burden by red chromatic shift (RCS). n = 3, error bars represent standard deviation, at time 0 and time 96 hours.

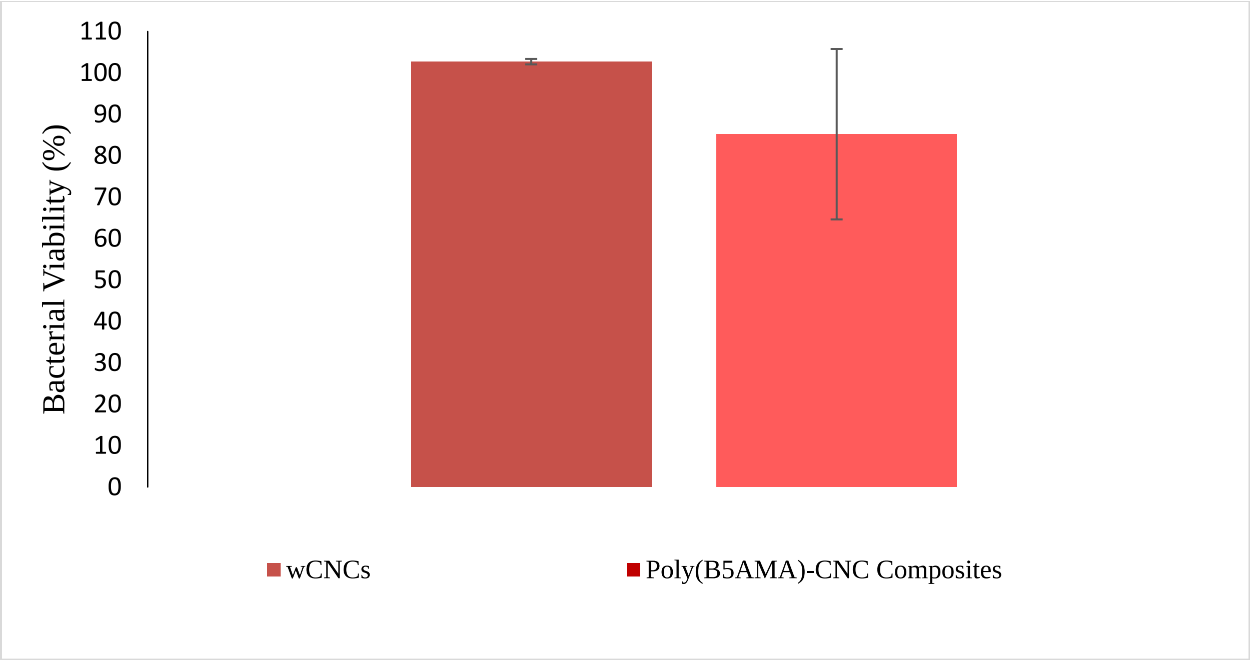

Figure S9: *E. coli* viability for samples treated with CNCs, and poly(B5AMA)-CNCs composites at concentrations of 16mg/mL for all samples. Error bars represent standard deviation, all values are calculated relative to untreated positive control which was taken as 100% viability.

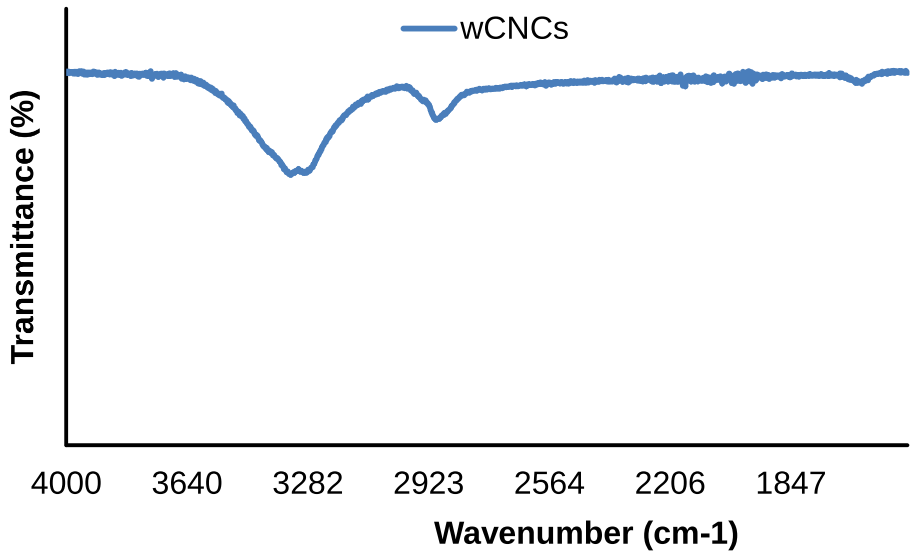

Figure S10: FTIR spectrum of bare CNCs.

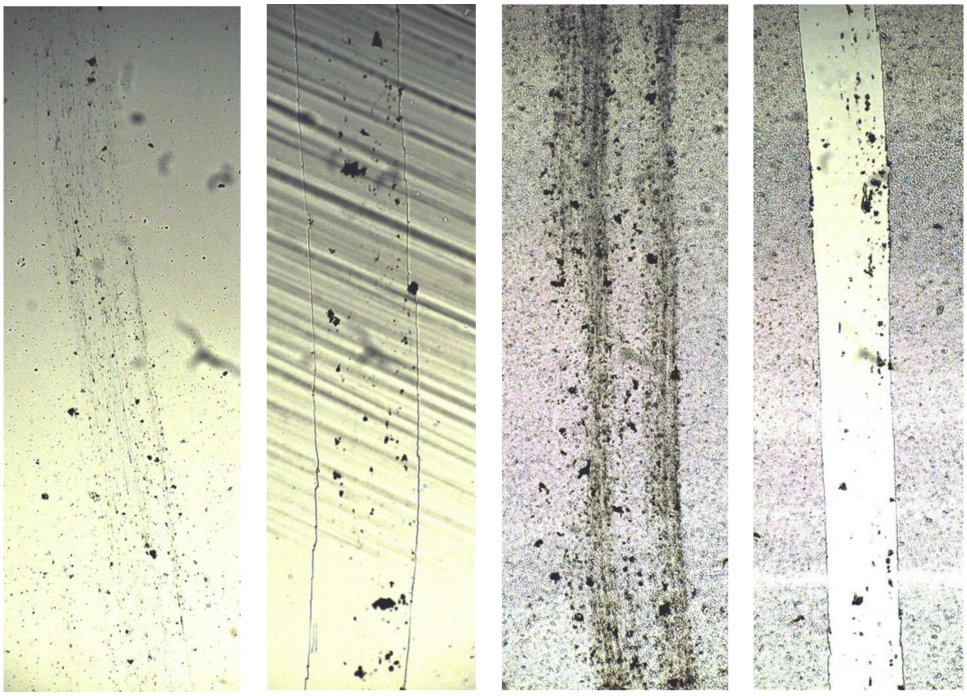

Figure S11: Light Microscope images of CNC Coated glass slide treated with 4B pencil (left most), CNC Coated glass slide treated with 2B pencil (center left), Poly(B5AMA)-CNC coated glass slide treated with B pencil (center right), Poly(B5AMA)-CNC coated glass slide treated with F pencil (right).
